# The statistical analysis of a clinical trial when a protocol amendment changed the inclusion criteria

**DOI:** 10.1186/1471-2288-8-16

**Published:** 2008-04-08

**Authors:** Christian Lösch, Markus Neuhäuser

**Affiliations:** 1 Institute for Medical Informatics, Biometry and Epidemiology, University of Duisburg-Essen, Hufelandstr. 55, 45122 Essen, Germany; 2 Department of Mathematics and Technique, RheinAhrCampus, Koblenz University of Applied Sciences, Südallee 2, 53424 Remagen, Germany

## Abstract

**Background:**

Sometimes, protocol amendments that change the inclusion and exclusion criteria are required in clinical trials. Then, the patient populations before and after the amendment may differ.

**Methods:**

We propose to perform separate statistical tests for the different phases, i.e. for the patients recruited before and after the amendment, and to combine the tests using Fisher's combination test. After a significant combination test a multiple testing procedure can be applied to identify the phase(s) to which a proof of efficacy refers. We assume that the amendment(s) are not based on any type of unblinded data. The proposed method is investigated within a simulation study.

**Results:**

The proposed combination approach is superior to the 'naïve' strategy to ignore the differences between the phases and pooling the data to perform just one statistical test. This superiority disappears when there are hardly any differences between the two phases.

**Conclusion:**

When one or more protocol amendments change the inclusion and exclusion criteria, one should realize that the populations may differ. In this case, separate tests for the different phases together with a combination test are a powerful method that can be applied in a variety of settings. The (first) amendment should specify the combination test to be applied in order to combine the different phases.

## Background

Protocol amendments are often necessary in clinical trials. Sometimes a change in the inclusion and/or exclusion criteria is required. There are various reasons for a change of the inclusion and exclusion criteria, some of them are mentioned in the ICH E9 guideline [1] and by Cleophas et al. [2]. On the one hand, newly emerging medical knowledge can be one reason especially for long-term trials, on the other hand, regular violations of entry criteria and too low recruitment rates could also make changes necessary. In any case, changes of the inclusion/exclusion criteria have to be described in a protocol amendment. Moreover, according to Cleophas et al. [2], the "amendment should also cover any statistical consequences ... and alterations to the planned statistical analysis".

When entry criteria are changed during the trial, the populations before and after the amendment may differ. Chow and Shao [3] presented an example of a placebo-controlled clinical trial in patients with asthma. Because patient enrolment was slow the inclusion criteria were relaxed. To be precise, according to the original protocol, patients with a baseline FEV_1 _(forced expiratory volume in liter per second) between 1.5 and 2.0 could be included. The first amendment extended this range up to an upper bound of 2.5, and the second amendment to an upper bound of 3.0. Please note that the change in FEV_1_, i.e. the difference between the value after treatment and that at baseline, was the primary endpoint in this study.

Other examples of amendments that change entry criteria were presented [4,5]. Svolba and Bauer's [4] example is a two-armed, long-term trial that investigated the time until relapse of cutaneous melanoma. In this study an amendment to the protocol increased the inclusion limit for cholesterol. Dubertret et al. [5] report the results of a phase III placebo-controlled trial in patients with moderate-to-severe plaque psoriasis. In this trial efalizumab, a humanized monoclonal antibody, was compared with placebo. During the study the protocol was amended to modify the inclusion criteria.

These examples show that amendments that relax or modify the entry criteria occur in different indications. Moreover, in the examples the entry criteria were changed once or at least twice. In practice, the inclusion and exclusion criteria are rarely changed very often.

As mentioned above, the actual patient population after an amendment may deviate from the originally targeted population [3]. For example, a modification of the acceptable range of baseline values can change the variance of the difference value after treatment minus baseline [6]. Usually, the difference in the populations before and after the amendment is ignored in the statistical analysis. As a consequence, the data are pooled; a procedure that can introduce a bias and can decrease the power of the study, maybe beneath the necessary power which was fixed when planning the study.

Chow and Shao [3] proposed a method that takes the potential differences in the populations before and after an amendment into account. The main idea is to divide the trial data according to the different treatment groups and phases. A new phase is started after each amendment. Thus, when there are *K *amendments *K *+ 1 phases result (*K *≥ 1). For each combination of group (T for treatment and C for control group) and phase (for phases 0, 1,..., *K*), a single value for the endpoint *y *and value(s) the predictor *x *(which may be of dimension greater than 1) are determined by e.g. computing the sample means. In the case of *K *amendments one gets the points $\left(x_{0}^{C},y_{0}^{C}),...,(x_{K}^{C},y_{K}^{C}\right)$ for the control group and $\left(x_{0}^{T},y_{0}^{T}),...,(x_{K}^{T},y_{K}^{T}\right)$ for the treatment group. Then, weighted linear regression analysis should be performed on the points for the control and treatment group, respectively.

As mentioned above, changing inclusion and exclusion criteria can change the target population. A change in the target population, however, can cause a change of the efficacy parameter. Therefore, we have *K *+ 1 possibly different null hypotheses, one for each phase. Let $\mu_{i}^{T}(\mu_{i}^{C})$ be the population mean of a normally distributed endpoint in phase *i *in the treatment (control) group. Then, the *i*-th null hypothesis is ${\text{H}}_{0}^{i}:\mu_{i}^{T}=\mu_{i}^{C}$, *i *= 0, 1,..., *K*. The global null hypothesis is the intersection of the different null hypotheses: ${\text{H}}_{\text{0}}{\text{:H}}_{0}^{0}\cap \ldots \cap {\text{H}}_{0}^{K}$. Note that even if efficacy is the same in all phases under the intersection null hypothesis, $\mu_{i}^{T}\neq \mu_{j}^{T}$ for *i *≠ *j *is possible as long as $\mu_{i}^{T}=\mu_{i}^{C}$ holds for every *i*, i.e. when the mean difference is constant over the phases, the population means may differ between the phases under H_0_. The variances are assumed to be equal for the two groups within each phase. However, there may be differences in variability between the different phases.

In case of one single amendment the procedure proposed by Chow and Shao [3] reduces to a weighted regression for two data points for the study and control group respectively. If one then decides to model the effect for the original target population (or the amended one) alone the procedure yields the maximum likelihood estimate for the mean for the first (second) population. One would probably not use this estimate to test the intersection hypothesis. For testing the intersection null, one would have to use some other contrast as briefly mentioned by Chow and Shao [3] on page 661.

As an alternative we propose a test procedure based on a combination test. We suggest analysing the subpopulations before and after the change, or, in general, the *K *+ 1 subpopulations, separately and then combining the *p*-values of the test statistics. We use Fisher's combination test, one of the methods that can be recommended according to an extensive simulation study [7]. This test uses that, under the null hypothesis, -2log(*p*_1 _⋯ *p*_*k*_) has a $\chi_{2K}^{2}$ – distribution if the *p*-values *p*_*i *_are independent [8]. When the intersection hypothesis *H*_0 _is true and the whole study is divided into two or more phases, then each ${\text{H}}_{0}^{i}$ should be true and the *p*-values *p*_*i *_are independent because each patient contributes to one *p*-value only. Furthermore, it is assumed here that the protocol amendment is independent from the *p*-values and hence cannot be based on any type of unblinded data. This limitation is further discussed below.

In practice, it may or may not be appropriate to test only the intersection null hypothesis, depending on the goal of the trial. Rejecting the intersection null hypothesis does not automatically permit to identify the population(s) where the treatment is efficient. In order to justify to which population (= phase) a proof of efficacy refers a multiple testing procedure can be used [9-11]. A multiple testing procedure that controls the multiple level *α *can easily implemented when Fisher's combination test is used as proposed above. In case of one single amendment, when the combination test rejects the intersection hypothesis, those individual null hypotheses ${\text{H}}_{0}^{i}$ (*i *= 0, 1) can be rejected for which the corresponding test gives a *p*-value not exceeding *α *[12] (page 1034).

## Methods

We performed a simulation study with normally distributed endpoints and one protocol change. This protocol change takes place when half of the patients are recruited (scenario 1) or when a third of the patients is recruited (scenario 2). After the protocol change the variance was inflated, thus the second phase had a larger variability. We assumed a two group setting with one group being the treatment group and the other being the control group.

The factor for the variance change in the second phase (variance inflation factor) was always equal for both groups. To be precise, we used the following variance inflation factors: 1 (i.e. no change), 1.5, 2, 2.5, and 3. The configurations of means for the simulations can be divided into four groups:

1. Investigation of the type I error rate: $\left(\mu_{0}^{T},\mu_{1}^{T},\mu_{0}^{C},\mu_{1}^{C}\right)$ = (0, 0, 0, 0).

2. Investigation of the power in the case of constant means within groups and with a constant non-zero between-groups mean difference *s*: $\left(\mu_{0}^{T},\mu_{1}^{T},\mu_{0}^{C},\mu_{1}^{C}\right)$ = (*s*, *s*, 0, 0), for the shift (i.e. the mean difference) *s *the values 0.05, 0.10, 0.15, 0.20, 0.25, 0.30, 0.40, 0.50, 0.60, 0.70, 0.75, 0.80, 0.85, 0.90, 0.95, and 1.00 were used.

3. Investigation of the power in the case of

a. non-constant means, i.e. within the two groups, means can differ between phases by $d=\mu_{1}^{T}-\mu_{0}^{T}=\mu_{1}^{C}-\mu_{0}^{C}\neq 0$ and simultaneously

b. a constant between-groups mean difference $s=\mu_{0}^{T}-\mu_{0}^{C}=\mu_{1}^{T}-\mu_{1}^{C}\neq 0$

leading to $\left(\mu_{0}^{T},\mu_{1}^{T},\mu_{0}^{C},\mu_{1}^{C}\right)$ = (*s*, *s *+ *d*, 0, *d*). For *s *as well as for *d *the values 0.1, 0.5, and 1 were used.

4. Investigation of the power in the case of non-constant means (i.e. within groups, means can differ between phases) and a non-constant between-groups mean difference: The following special cases are presented in this paper: $\left(\mu_{0}^{T},\mu_{1}^{T},\mu_{0}^{C},\mu_{1}^{C}\right)$ = (0.5, 0.2, 0, 0) for scenario 1 and $\left(\mu_{0}^{T},\mu_{1}^{T},\mu_{0}^{C},\mu_{1}^{C}\right)$ = (0.7, 0.2, 0, 0) for scenario 2.

In addition, we investigated the effect of a decrease in variance. To be precise, we simulated data with a shift of 0.1, 0.5, or 1, respectively, and a reduction of the standard deviation to 0.25, 0.5 and 0.75, respectively.

We used two possible strategies of evaluating the data. The first strategy is simply pooling the data and performing a one-sided *t*-test with the assumption of homoscedasticity (which is in fact fulfilled). The second analysis is to perform a one-sided *t*-test for each of the two phases separately and then using Fisher's combination test to obtain an overall result. It should be noted that the combination of *p*-values across populations is essentially a meta-analytic method. Regarding the combination test we present results for testing the intersection hypothesis as well as for identifying efficacy in at least one population. The latter one will be abbreviated as "com & one" in the figures. For all strategies the (empirical) *α*-level of the simulation was determined as well as the (empirical) power of the test. All simulations were performed with SAS (version 9.1) and 10 000 simulation runs, except for estimating the type I error rate which is based on 100 000 simulation runs. We set *α *= 0.05.

We use the following notation:

$n_{b}^{C}$ : number of patients in control group before the amendment,

$n_{b}^{T}$ : number of patients in treatment group before the amendment,

$n_{a}^{C}$ : number of patients in control group after the amendment, and

$n_{a}^{T}$ : number of patients in treatment group after the amendment.

In the first scenario all four sample sizes $n_{b}^{C}$, $n_{b}^{T}$, $n_{a}^{C}$ and $n_{a}^{T}$ are 50. In the second scenario, we have $n_{b}^{C}$ = $n_{b}^{T}$ = 25 and $n_{a}^{C}$ = $n_{a}^{T}$ = 50.

## Results

### Simulation study

**Figure 1:**
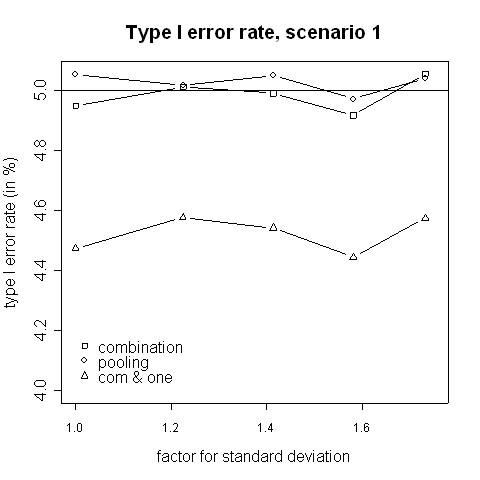
**Type I error rate, scenario 1**. Type I error rate of the pooling and the combination strategies for inflation of the standard deviation in scenario 1 (i.e. amendment is implemented when half of the patients have been accrued).

**Figure 2:**
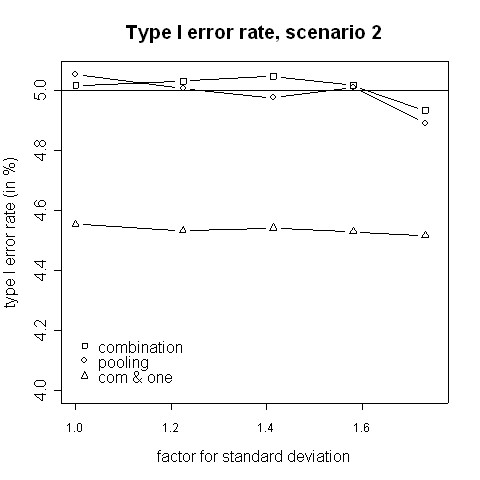
**Type I error rate, scenario 2**. Type I error rate of the pooling and the combination strategies for inflation of the standard deviation in scenario 2 (i.e. amendment is implemented when a third of the patients have been accrued).

Both, the pooling and the combination strategy must hold the type I error rate because the tests' requirements are fulfilled. The simulated type I error rates are presented in Figures 1 and 2. Slight deviations from 5% are caused by simulation errors. In contrast, when in addition to a significant combination test the rejection of at least one ${\text{H}}_{0}^{i}$ is required, the size is reduced to approximately 0.045. However, it can be expected that the applied closed testing procedure is conservative.

**Figure 3:**
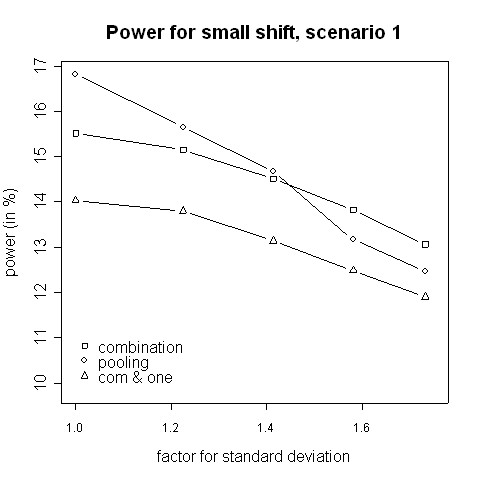
**Power for small shift, scenario 1**. Power of the pooling and the combination strategies for inflation of the standard deviation in scenario 1 in the case of a small shift (0.1).

**Figure 4:**
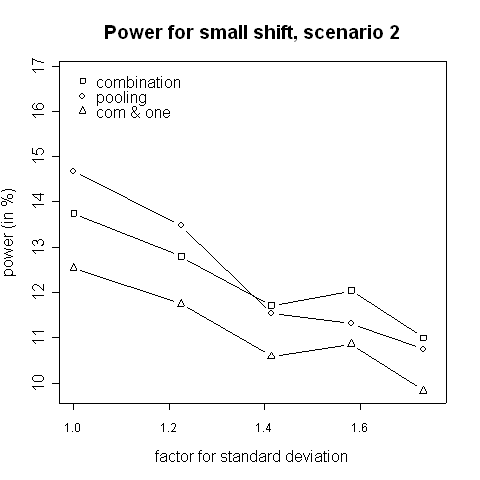
**Power for small shift, scenario 2**. Power of the pooling and the combination strategies for inflation of the standard deviation in scenario 2 in the case of a small shift (0.1).

**Figure 5:**
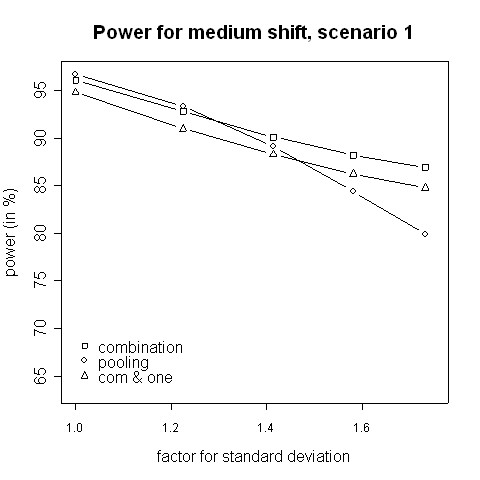
**Power for medium shift, scenario 1**. Power of the pooling and the combination strategies for inflation of the standard deviation in scenario 1 in the case of a medium shift (0.5).

**Figure 6:**
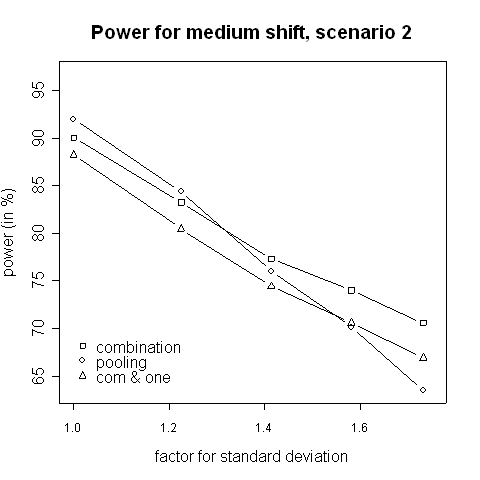
**Power for medium shift, scenario 2**. Power of the pooling and the combination strategies for inflation of the standard deviation in scenario 2 in the case of a medium shift (0.5).

With regard to power, the results from the simulations indicate that for small variance inflation factors simply pooling the data and ignoring the fact that there are two different variances yields a higher power than using the combination strategy (Fig. 3, 4). In that case there is hardly any difference between the two phases, therefore one test with pooled data is more powerful than applying a combination test [13]. However, when the standard deviation is distinctly higher in the second phase (e.g. $\sqrt{2.5}$ ≈ 1.58 times higher) the combination procedure is either as good or it has a higher power than pooling. The inflation factor needed to let the combination procedure be more powerful than the pooling procedure was found to be between 1.4 and 1.6 (Figures 3 to 6). Such an inflation is not uncommon in real trials (see the example given by Koch [10]).

**Figure 7:**
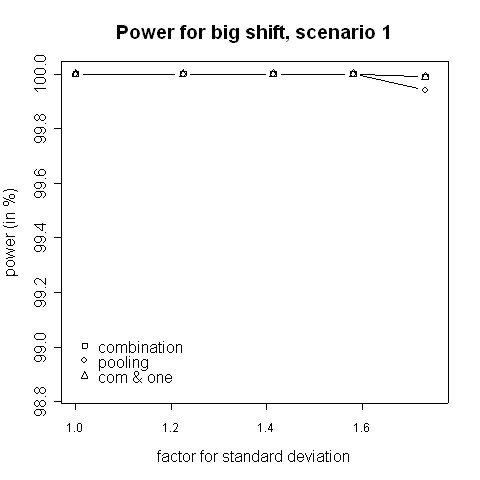
**Power for big shift, scenario 1**. Power of the pooling and the combination strategies for inflation of the standard deviation in scenario 1 in the case of a big shift (1.0).

**Figure 8:**
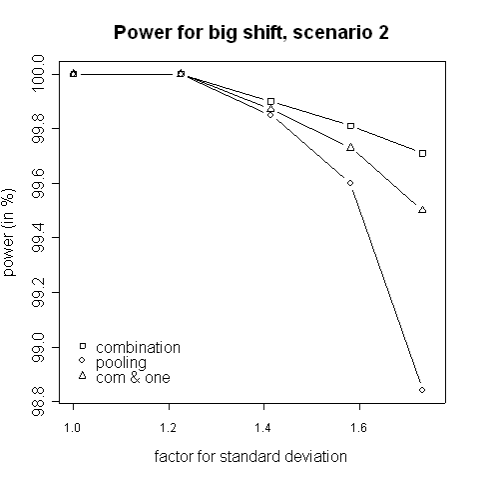
**Power for big shift, scenario 2**. Power of the pooling and the combination strategies for inflation of the standard deviation in scenario 2 in the case of a big shift (1.0).

Consider the absolute difference *δ*_*a *_= *β*_*c *_- *β*_*p *_of the power *β*_*c *_of the combination approach and the power *β*_*p *_of the pooling approach: for very small shifts (e.g. 0.05 to 0.2) *δ*_*a *_was found to be between -2.41% and 3.01% (Figures 3, 4). For very big shifts (0.8 to 1.0) the power difference was between -0.05% and 3.07% (Figures 7, 8). A bigger difference can be seen at medium shifts (0.2 to 0.75) where it ranges from -3.35% to 7.11% (Figures 5, 6). Nevertheless, a trend can be observed for the absolute difference *δ*_*a *_and relative difference *δ*_*r *_= (*β*_*c *_- *β*_*p*_)/*β*_*c *_compared to the variance inflation factor. In case of small factors the difference is often negative, so pooling may be preferable. With increasing factors, however, the differences are more and more positive, i.e. the combination strategy is more powerful.

When not only a significant intersection hypothesis, but also efficacy in at least one population is required, the power is smaller. Figures 3 to 8 also display the extent of the reduction in power. According to our simulations this reduction is not very large and it is noteworthy that the combination strategy with the requirement to reject at least one ${\text{H}}_{0}^{i}$ can be more powerful than pooling (see Figures 5 to 8).

**Figure 9:**
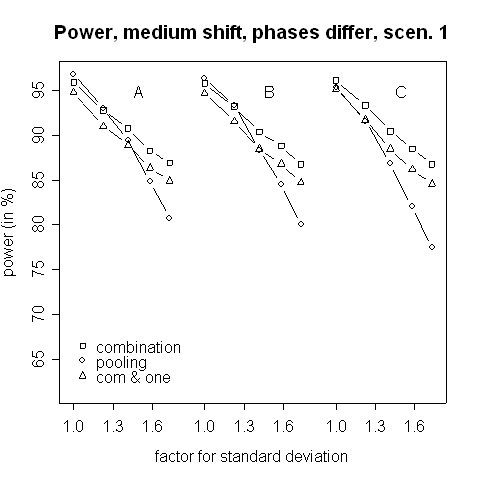
**Power for medium shift, phases differ, scenario 1**. Power of the pooling and the combination strategies for inflation of the standard deviation in scenario 1 in the case of a medium shift (0.5). Within each group, means differ between the two phases by (A) 0.1, (B) 0.5 and (C) 1.0.

**Figure 10:**
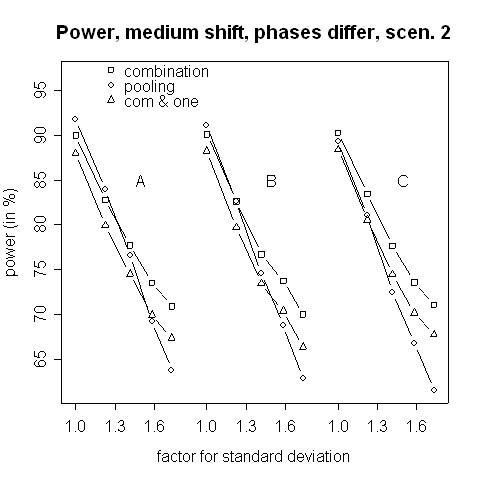
**Power for medium shift, phases differ, scenario 2**. Power of the pooling and the combination strategies for inflation of the standard deviation in scenario 2 in the case of a medium shift (0.5). Within each group, means differ between the two phases by (A) 0.1, (B) 0.5 and (C) 1.0.

The power in case of $\mu_{0}^{T}-\mu_{1}^{T}=\mu_{0}^{C}-\mu_{1}^{C}\neq 0$ is shown in Figures 9 and 10. Here, the combination procedure can be distinctly more powerful than pooling: there is hardly any difference in power when the standard deviation is the same in both phases. But the larger the variance inflation is the larger is the superiority of the combination strategy. The difference is so big that even the combination strategy with the additional requirement to reject at least one $H_{0}^{i}$ is more powerful that pooling as far as the variance inflation is approximately 1.5 or higher. Regarding Figures 9 and 10, please note that the stagewise *t*-tests are independent of the within-group means and depend on the mean differences only. Thus, the power curves A, B, and C of the combination test are theoretically equal and differ due to simulation errors only. Furthermore, the dependence of the overall *t*-test on the different within-group means is only caused by an inflation of the standard error estimate.

**Figure 11:**
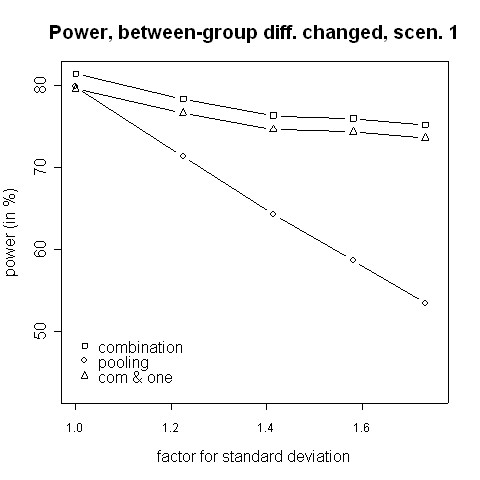
**Power for changing between-group differences, scenario 1**. Power of the pooling and the combination strategies for inflation of the standard deviation in scenario 1 in the case of a decrease of the between-groups mean difference from 0.5 to 0.2.

**Figure 12:**
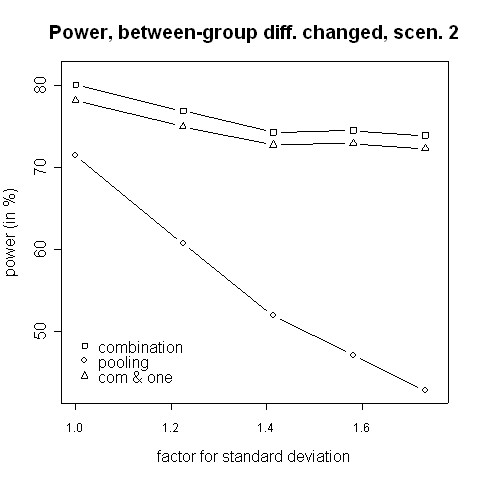
**Power for changing between-group differences, scenario 2**. Power of the pooling and the combination strategies for inflation of the standard deviation in scenario 2 in the case of a decrease of the between-groups mean difference from 0.7 to 0.2.

Figures 11 and 12 show simulation results for the case where the treatment effect is changed by the change of the inclusion and exclusion criteria. The amendment might extend the range of possible patients, in particular when recruitment rates are low. In the then more heterogeneous population a lower treatment effect can be expected. Therefore, we considered the case of a lower treatment effect after the amendment. The combination test works well in this situation, even when efficacy in at least one phase is required.

As mentioned above, we additionally investigated a decreased variability in the second phase. For symmetry reasons, the results are analogous and, therefore, not presented.

## Discussion and conclusion

For small variance inflation in the second phase pooling seems to be better than the combination strategy. This is quite obvious because the populations before and after the amendment hardly differ and, consequently, pooling is admissible. For very small shifts and very big shifts no big difference was found between the pooling strategy and the combination test, even in the presence of a bigger variance in the second phase. In the case of a small shift both procedures have a very low power, so the small difference in power may be not important. In the case of a big shift both procedures have a very high power, and a small difference may be unimportant, too. However, in the case of a medium shift the combination strategy was noticeably more powerful than pooling, the difference in power was up to 7.1%.

When the variability of the second phase is reduced a moderate or large reduction leads analogously to superiority of the combination strategy. Furthermore, the combination strategy is particularly superior when $\mu_{0}^{T}-\mu_{1}^{T}=\mu_{0}^{C}-\mu_{1}^{C}\neq 0$. Thus, the simulation study indicates that the combination strategy is superior when a substantial difference in variability between the two phases exists. In addition, within a closed testing procedure it can be tested after a significant combination test whether there is a significant efficacy within the separate phases.

Moreover, a combination test has the advantage that it can also be applied for any other types of data such as e.g. non-normal, count or time to event data. The regression approach [3] is more limited, and the pooling approach may cause problems such as Simpson's paradox when pooling count data from different phases.

We consider the case that an amendment changes entry criteria because, for example, they are regularly violated, because of low recruitment rates or because of external information such as accumulating medical knowledge in long-term trials. Of course, it is preferable to avoid amendments during the trial. However, sometimes they are necessary and if necessary, an amendment should change the entry criteria as less as possible. As mentioned above in the Introduction the amendment should also cover the statistical consequences [2]. Thus, the amendment should specify to separately analyse the different phases and to combine the *p*-values with (e.g.) Fisher's product test.

We do not consider data dependent adaptations. Therefore, the amendment must be independent from the *p*-values and hence cannot be based on any type of unblinded data. This limitation also applies to the approach of Chow and Shao [3]. It should be noted that this limitation to non-data driven amendments can be avoided by using the conditional error rate principle of Müller and Schäfer [14].

In our simulation study we assumed that the variance of both treatment arms was equally affected by the amendment. Different inflations of variances and/or location shifts may increase the differences between the two phases. These differences are probably much more problematic for the pooling than the combination strategy.

The populations before and after the amendment can differ. Thus, one may argue for performing a test of interaction between phase (pre versus post-amendment) and treatment. The multiple testing procedure mentioned above, that can be used to investigate to which phase a proof of efficacy refers, would be especially important when a treatment by amendment interaction exists. However, we recommend performing the interaction test in an exploratory manner only.

The focus of our paper in on hypothesis testing. We have not addressed issues related to estimation of the treatment effect which usually are also important in clinical trials. Regarding estimation we refer to Brannath et al. [15]. The methods presented in their overview [15] may also be applied when there is no data dependent adaptation after stage 1.

In summary, if there has been an amendment which affected the entry criteria, we recommend using the combination strategy. This suggestion holds in particular when the variability of the endpoint may be influenced by the amendment. Furthermore, the proposed procedure is easy to apply and flexible as well.

## Competing interests

The author(s) declare that they have no competing interests.

## Authors' contributions

CL performed the simulation study. Both authors designed this study, drafted parts of the manuscript, read and approved the final manuscript.

## Pre-publication history

The pre-publication history for this paper can be accessed here:


